# Supplementary material for: The impact of moderate-intensity basketball intervention on the physical self-esteem and school adjustment of first-year high school students
Source: PeerJ. 2024 Sep 18;12:e17941. doi: 10.7717/peerj.17941 (PMC11416090; doi:10.7717/peerj.17941)
Supplement: Supplemental Information 2 [file peerj-12-17941-s002.docx]

Variable assignment table

| Study variables | Assignment |
| --- | --- |
| group | 1=Control group，2=Experimental groups |
| Gender | 1=Male，2=Female |
| Physical self-esteem | 1=completely inconsistent, 2=relatively inconsistent, 3=relatively consistent, 4=completely consistent |
| School adaptation | 1=completely inconsistent, 2=relatively inconsistent, 3=uncertain, 4=relatively consistent, 5=completely consistent |
